# Smokers Are More Likely to Smoke More after the COVID-19 California Lockdown Order

**DOI:** 10.3390/ijerph18052582

**Published:** 2021-03-05

**Authors:** Mariaelena Gonzalez, Anna E. Epperson, Bonnie Halpern-Felsher, Deanna M. Halliday, Anna V. Song

**Affiliations:** 1 Nicotine and Cannabis Policy Center, University of California, Merced, CA 95343, USA; aepperson@ucmerced.edu (A.E.E.); bonnieh@stanford.edu (B.H.-F.); dhalliday@ucmerced.edu (D.M.H.); asong5@ucmerced.edu (A.V.S.); 2 Department of Public Health, School of Social Sciences, Humanities, and Arts, University of California, Merced, CA 95343, USA; 3 Department of Psychological Sciences, School of Social Sciences, Humanities, and Arts, University of California, Merced, CA 95343, USA; 4 Division of Adolescent Medicine, Department of Pediatrics, Stanford University School of Medicine, Stanford, CA 94304, USA

**Keywords:** COVID-19, tobacco products, cigarette smoking, vaping, electronic nicotine delivery systems, California

## Abstract

To determine if cigarette smoking, electronic cigarette use, and rate of consumption of these products differed before and after a pandemic lockdown order, two convenience samples of adults in Central California were recruited and surveyed before (March 2020) and after (May 2020) COVID-19 lockdown orders were implemented in California (*n* = 2571). Multivariable logistic and negative binomial regression models tested the association between adults recruited pre- or post-California lockdown and past month cigarette use, past month electronic cigarette use, past month cigarette consumption, and past month e-cigarette consumption among current users, controlling for demographic differences. Adults pre- and post-lockdown had equal odds of using cigarettes during the past month. Cigarette users who responded post-lockdown had higher cigarette consumption rates compared to cigarette users who responded pre-lockdown (IRR = 1.13, 95% CI = 1.15, 1.23). Adults who responded post-lockdown had lower odds of using electronic cigarettes during the past month compared to participants surveyed before the order (OR = 0.66, 95% CI: 0.55, 0.78). Cigarette users may be using more cigarettes during the state mandated lockdown. Possible causes for this increase in cigarette use may include increased stress, the change in workplace smokefree protections coverage, and increased opportunities for smoking or vaping.

## 1. Introduction

Tobacco users are at higher risk for having more severe COVID-19 outcomes [1,2,3,4,5,6,7]. As a result, the COVID-19 pandemic brings urgency to tobacco control efforts to reduce tobacco consumption [1], educate tobacco users of their increased risks, and strongly encourage all tobacco cessation. However, other stressors resulting from the pandemic and related lockdown orders, such as increased anxiety, social isolation, and economic concerns, may have increased. These stressors in turn may lead to increased initiation of tobacco use by non-users and/or increased product use among current users [1,8]. As a result, it is important to understand how pandemic-related lockdown orders have impacted tobacco-related behavior.

Research on the impact of COVID-19 on adult tobacco-related behaviors is emerging [9,10,11,12,13]. One study examined post-lockdown data on adult tobacco use patterns in 5 countries, including the U.S., during the COVID-19 pandemic and found that electronic cigarette (e-cigarette) use marginally increased in all five countries. An increase in cigarette smoking was only found among respondents from India and Italy [9]. Another study conducted in Italy also found that e-cigarette consumption increased during lockdown and that exclusive cigarette smoking decreased [10,12,14]. A study conducted in the U.S. found that adolescents and young adults reported a decrease in e-cigarette use during the lockdown [13]. Taken together, these studies indicate that the pandemic-related lockdown may have contributed to an increase in e-cigarette use among adults; findings are less clear about the impact on cigarette smoking. In addition, these inferences are based on retrospective reports of pre-lockdown tobacco behaviors after lockdowns were implemented. To date, no study has compared data collected before and after a COVID-19 lockdown to assess how COVID-19 and the related lockdown might affect tobacco use and consumption amount among adults.

We examined two ways in which pandemic-related lockdown orders may have impacted the use of tobacco products among a sample of adults living in Central California before and after the state mandated lockdown order was issued on 19 March 2020: (1) changes in past month use of traditional cigarettes and e-cigarettes, and (2) changes in cigarette and e-cigarette consumption rates. Past month use is an indicator of the number of current users in the community. Consumption rates are an indicator of whether current users are increasing or decreasing their usage of cigarettes or e-cigarettes. In this regard, our study seeks to understand lockdown impacts at the community level (number of users), as well as at the individual user level (intake amount). Central California represents a vast area that includes urban, suburban, and rural communities. Moreover, this region is home to a diverse cross-section of racial/ethnic and socioeconomic status groups. Like many regions across the U.S., Central California has historically faced economic challenges that have translated to a weaker health infrastructure, including mental health and tobacco cessation services, translating to higher tobacco use and potential for greater susceptibility and severity of the COVID-19 virus [15,16,17]. Study findings may provide insight on how tobacco-related product use may change in response to significant social shifts brought by the COVID-19 pandemic.

## 2. Methods

### 2.1. Participants and Design

The convenience sample consisted of 2571 adults (1510 surveyed in early March 2020 and 1061 in May 2020). Participants resided within an 11-county region in Central California. All participants were recruited via focused advertisements on social media that targeted any resident of the 11-county area. Interested participants were directed to an online screening survey deployed via Qualtrics, where participants were screened for age and county of residence. Eligibility criteria included being 18 years or older, living in the 11-county area, and being English literate. If eligible, participants were directed to the full survey; participants were compensated a gift certificate of $5 for completing the survey. To assure validity of responses, participants whose survey response time did not fall within two standard deviations of the average survey completion time were excluded from analysis. While there have been many cutoff points used in the literature [18], we chose two standard deviations as a reasonable cutoff point. 

### 2.2. Measures

#### 2.2.1. Dependent Variables

Participants were classified as a past-30-day (i.e., past month) cigarette user if they had used a cigarette at least once during the last 30 days. Past-30-day e-cigarette users were classified as participants who reported that they had used an e-cigarette product at least once during the past 30 days. In order to compute a respondent’s cigarette consumption rate that accounted for both number of days smoked (0–30) and number of cigarettes smoked per day (0–100 plus), we modified a measure used by Johnson and colleagues [19], where a respondent’s cigarette consumption rate is calculated as the number of cigarettes consumed per day, multiplied by the number of days on which a cigarette was smoked. Historically in the literature, cigarette consumption has been measured in two ways—number of days tobacco was consumed during the month (e.g., someday and everyday smoking) and the average number of cigarettes consumed per day (e.g., chipper, light, half-pack, etc.). As a result, it has been difficult to compare these two types of consumption patterns. Calculating the number of cigarettes consumed in a month (average number of cigarettes consumed per day × number of days cigarettes were used in a month) allowed us to estimate a measure which takes into account both daily cigarette smoking and the number of days in a month cigarettes were smoked. We used a similar measure to calculate e-cigarette consumption. Respondents were asked to estimate the number of times per day that they used e-cigarettes as 1–10, 11–20, 21–20, or 31 times or more. In order to estimate an e-cigarette usage rate similar to the cigarette rate, the reported number of times per day a respondent used an e-cigarette was set to the lowest value of the category (1, 11, 21, and 31) and multiplied by the number of days of the month on which a user reported using an e-cigarette. This allowed us to calculate the number of times respondents reported using e-cigarettes in a given month, allowing us a past-month comparison of use which is an estimated indicator of differences in consumption levels.

#### 2.2.2. Independent Variables

Participants were categorized as responding pre-lockdown if they responded to the survey in early March 2020 and post-lockdown if they responded in May 2020. Participants were asked basic demographic questions (sex [male/female], age [18–25 years/≥26 years], race/ethnicity, and socioeconomic status) and whether they were living with a partner (yes/no). In order to categorize racial/ethnic affiliation, participants were first asked if they identified as Hispanic/Latino. Participants were then asked to select which other racial/ethnic groups they identified with African American/Black, American Indian/Alaska Native, Caucasian/White, East Asian (e.g., Chinese, Korean, Japanese), Middle Eastern (e.g., Afghani, Syrian, Persian, Yemeni), Pacific Islander/Native Hawaiian, Southeast Asian (e.g., Filipino, Vietnamese, Hmong, Mein), South Asian (e.g., Indian, Pakistani, Sri Lankan), or Other. Respondents’ race/ethnicity was classified as Hispanic/Latino (of any race), non-Hispanic (NH) White (no other race/ethnicity), NH African American/Black (no other race/ethnicity), and NH Other (including racial/ethnic categories not previously listed and mixed-race participants who did not identify as Hispanic/Latino). Two measures were used for socioeconomic status (SES): participants were asked about their highest level of education attained (high school diploma or GED or less/some college/college degree or higher) and household income level which was divided into four-levels (≤USD 50,000, USD 51,000–USD 75,000, USD 76,000–USD 100,000, and ≥USD 101,000).

### 2.3. Statistical Analysis

Two multivariable binary logistic regressions were run to explore the relationship between participants surveyed before and after the lockdown order and (1) past 30-day use of cigarettes and (2) past 30-day use of e-cigarettes. To test whether or not pre- and post-lockdown respondents varied demographically, we used a chi-square test (see Table 1). The final statistical models adjusted for demographic differences between the two groups (sex, age, race/ethnicity, living with a partner, income, and education), as well as for income [20]. The relationship between being interviewed before or after lockdown and (1) cigarette consumption among cigarette users and (2) e-cigarette usage among e-cigarette users was analyzed via two negative binomial regressions, from which incidence rate ratios (IRR) were calculated. In the context of our study, the IRR is a comparison of incidence rates between two groups (e.g., pre- versus post-lockdown respondents) during the study period. An analysis of cigarette users, using a chi-squared test (*p* ≤ 0.05), indicated that individuals who responded pre/post-COVID varied demographically on the basis of age, sex, race/ethnicity, living with a partner, education, and income. As a result, the final models pertaining to cigarette use were adjusted for all these factors. An analysis of e-cigarette users (using a chi-squared test, *p* < 0.05) indicated pre- and post-COVID respondents varied demographically on the basis of sex, age, race/ethnicity, living with a partner, and education. As a result, the models pertaining to e-cigarette use were adjusted for these factors, and for income, as income has been shown to be correlated with e-cigarette use [21]. Cases with missing data were listwise deleted. All analyses were conducted with STATA version 15.

## 3. Results

### 3.1. Study Samples

Participants were primarily female (64.72%, Table 1), between the ages of 18 and 25 years old (78.26%), NH White (66.75%) or Hispanic/Latino (18.79%), and had some college education (31.66%) or a bachelor’s degree or higher (50.10%). Over half the sample was located in a household where the income level fell below USD 76,000 (USD 0–USD 50,000 = 26.33%, USD 51,000–USD 75,000 = 32.83%) and were living with a partner (65.52%). Our sample primarily contained smokers who reported consuming over half a pack of cigarettes per day, as the majority of participants reported past 30-day cigarette use (85.56%) or past 30-day e-cigarette use (55.93%), and the mean number of cigarettes consumed per day was 12.31 (SD = 8.32).

### 3.2. Use of Cigarettes Was Higher Post-Lockdown, There Were Fewer E-Cigarette Users Post-Lockdown

Participants surveyed pre- and post-lockdown had equal odds of smoking cigarettes (OR = 0.96, 95% CI: 0.75, 1.23; Table 2) during the last 30-days (holding other variables constant in the model). Participants surveyed post-lockdown were estimated to have a higher incidence rate of cigarette consumption over the last 30 days (IRR = 1.13, 95% CI: 1.05, 1.23), compared to participants surveyed pre-lockdown (holding other variables constant in the model). All estimates were obtained after adjusting for sex, age, race/ethnicity, living with a partner, income, and education.

Participants who responded post-lockdown had lower odds of using e-cigarettes (OR = 0.66, 95% CI: 0.55, 0.78) during the past month compared to participants surveyed pre-lockdown (holding other variables constant in the model). There was no significant difference in the incidence rate for e-cigarette consumption over the last 30 days (IRR = 1.03, 95% CI = 0.90, 1.18) for participants surveyed post-lockdown versus pre-lockdown (holding other variables constant in the model). Models controlled for sex, age, race/ethnicity, living with a partner, income, and education.

## 4. Discussion

The COVID-19 pandemic lockdown in California was not associated with an increase in the number of individuals who used cigarettes or e-cigarettes. Indeed, the odds of being an e-cigarette user was lower post-lockdown than pre-lockdown. However, among current cigarette users, the lockdown was associated with an increase in cigarette consumption.

Many public health and healthcare professionals have worried that stress and isolation during the COVID-19 pandemic may lead to, or exacerbate, addictive behaviors [1,22,23,24]. This is especially concerning for regions that lack mental health infrastructure. The counties located in our 11-county area of surveillance have some of the lowest per capita ratio of behavior health professionals to population of all regions in California and the highest percentage of adults with serious mental disorders prior to the onset of the COVID-19 pandemic [15,25]. Similarly, prior to the onset of the pandemic, many rural regions had higher rates of poverty, substance use disorder, mental health problems, and suicide rates, but lower access to behavioral health services [26,27,28], indicating high levels of unmet need. Our analyses indicate that concerns regarding an increase in tobacco-related behaviors in regions with high levels of unmet need are warranted. It is imperative to bolster behavioral health and cessation resources in these communities where treatment is already difficult to attain, lest these areas become more marginalized due to COVID-19 related increases in mental and substance use disorders in areas where treatment is difficult to obtain.

When considering this increase in cigarette consumption, it should be noted that stress and other mental health issues may not be the only mechanism at play. Another factor potentially contributing to the increase in cigarette consumption concerns the impact of workplace smokefree policies. Most indoor workplaces in the U.S. have banned indoor smoking, and these smokefree workplace laws are associated with reduced smoking among workers [29,30,31]. Many cigarette users may have limited their consumption of cigarettes during worktime hours (e.g., scheduled smoking around work/break schedules). By being at home during the lockdown, the protective mechanisms of smokefree laws and the co-occurring limitation on times during which consumption can occur during working hours have changed for many current cigarette users. Prior to the pandemic, much of the discussion on smokefree areas had focused on bringing smokefree policies to communities that lack these protections [32,33]. However, the pandemic underscores how important these protections are. COVID-19 lockdowns may have mitigated the protections smokefree policies conferred to employees and communities, since most people were forced to shelter-in-place during the lockdown where smokefree policies often do not apply. This gap in protection and potential link to increases in smoking behavior also underscores the importance of smokefree policies in multi-unit housing in order to protect residents from secondhand smoke exposure.

Unlike previous adult studies that demonstrated an increase in respondents using e-cigarettes [9], the current study found that fewer participants surveyed post-lockdown reported using e-cigarettes in the past 30 days. A similar decrease in e-cigarette use was found among 13–24-year-old individuals in the U.S. [13]. The difference in findings may not be related to the ongoing pandemic, but to regional differences. In summer 2019, the U.S., including communities in California, experienced an outbreak of e-cigarette or vaping associated lung injury (EVALI) [34]. Central California was one of the first to see cases of EVALI, potentially heightening the awareness of the EVALI crisis among the community and thereby possibly reducing use. Additionally, a study of youth and young adults indicated that many e-cigarette users had difficulty with accessing product retailers during lockdown [13]. This difficulty in product access may also explain differences in findings between prior studies and the current study.

While literature on tobacco use during COVID-19 is increasing, it is important to clearly delineate community-level effects vs. individual-level effects. Our study indicates that while the number of users may be down, the pandemic does have an effect on tobacco usage, particularly as our study points to current cigarette users increasing their consumption rates.

Similar to other research, we found in our whole sample (pre- and post-pandemic respondents combined) that women and Hispanic/Latinos had lower odds of using a cigarette in the last 30 days, while adults 18–25 years old were more likely to use e-cigarettes than individuals over 25 years old [35,36,37,38,39]. We also found that low-income individuals had lower odds of reporting past 30-day cigarette use. One possible explanation for the observation that low-income individuals have lower odds of smoking is that around 80% of Hispanic/Latinos in the sample reported making less than USD 101,000, and Hispanic/Latinos have lower smoking rates than NH Whites [38,39]. In this regard, our observation that lower income respondents smoked less may be driven by the fact that most of the low SES respondents were also Hispanic/Latino. Another possible explanation is lack of access to tobacco retail outlets for low-income individuals in California. As previously mentioned, a prior study has found that many youth who were e-cigarette users had difficulty with accessing product retailers during the COVID-19 lockdown [13], and a similar issue may have occurred regarding cigarette retailers. Finally, low-income individuals may have had less excess income to spend on tobacco during COVID-19, and thus had an incentive to smoke less or quit during the economic recession that began in California during February-March 2020 [40].

## 5. Limitations

This study uses a convenience sample of participants recruited from social media and not a random sample, which impacts generalizability of study findings. However, the counties surveyed in the current study are akin to regions across the U.S., given their higher smoking rates and mix of rural, suburban, and urban populations. Additionally, this sample contains significantly more tobacco users than the general population of the region. However, this inadvertent oversample allows for a better understanding of tobacco user behavior. The oversampling of tobacco users may have occurred in response to the fact that our online ads had the word “nicotine” in the title, which may have made the study more attractive to smokers. The data are cross-sectional, and thus findings do not represent changes in a particular participant’s behavior, but rather differences at the community level; no causal inference can be made. Furthermore, the data cannot make inferences on the mechanism by which these pre-post differences occurred. The data are also subject to the limitations of self-reported data and the biases of recall. Respondents pre- and post-lockdown differed on some demographic factors, which was potentially due to the fact that participants who would not otherwise participate in surveys were more available during lockdown given confinement to home. While the analyses controlled for demographic variables that differed between respondents collected in March and May, there may be other underlying factors, such as perceived stress, social support, or other unobserved pandemic-related factors that could also possibly explain the differences in tobacco use between pre- and post-lockdown respondents. However, our primary goal was to understand how one major event in time might be related to community-level behavior and health.

## 6. Conclusions

Our findings indicate a shift in tobacco consumption at the community level in Central California post-COVID-19-related lockdown. While public health attention has been focused on a possible increase in addictive behaviors due to pandemic-related social and economic stress, another possible contributing factor could be that cigarette users were removed from protective indoor smokefree workplace laws and have increased time for cigarette smoking due to changes in working schedules. Interventions for quitting or reducing consumption need to take this into account to address the needs of tobacco users during the pandemic.

## Figures and Tables

**Table 1:** Sample characteristics, *n* = 2571.

Measures	Pre-Lockdown *n*(%)/M(SD) ^a^	Post-Lockdown *n*(%)/M(SD) ^a^	Total ^a^
*n*	1510 (58.73%)	1061 (41.27%)	2571
Any cigarette 30-day use	1270 (84.11%)	904 (85.20%)	2174 (85.56%)
Any e-cigarette 30-day use	878 (58.15%)	560 (52.78%)	1438 (55.93%)
Number of cigarettes smoked per day	12.99 (8.91)	11.81 (7.84)	12.31 (8.32)
Number of cigarettes consumed over past month	216.52 (182.48)	248.20 (201.00)	299.94 (191.14)
Number of times e-cigarettes were consumed in past month	106.56 (139.65)	108.66 (139.93)	107.38 (139.71)
Female *	1016 (67.28%)	648 (61.07%)	1664 (64.72%)
18–25 years old *	230 (15.23%)	297 (27.99%)	2012 (78.26%)
Race/ethnicity *			
Hispanic/Latino	318 (21.06%)	165 (15.55%)	483 (81.79%)
NH White	1008 (66.75%)	721 (67.95%)	1729 (67.25%)
NH African-American/Black	81 (5.38%)	102 (9.61%)	183 (7.12%)
NH Other	103 (6.82%)	73 (6.68%)	176 (6.85%)
Lives with partner *	944 (62.52%)	556 (52.40%)	1500 (58.34%)
Education *			
High school/GED or less	206 (13.64%)	214 (20.17%)	420 (16.34%)
Some college	566 (37.48%)	248 (23.37%)	814 (31.66%)
College degree or higher	734 (48.61%)	554 (52.21%)	1288 (50.10%)
Income			
USD 0–USD 50,000	401 (26.56%)	276 (26.301%)	677 (26.33%)
USD 51,000–USD 75,000	494 (32.72%)	350 (32.99%)	844 (32.83%)
USD 76,000–USD 100,000	382 (25.30%)	284 (26.77%)	341 (23.51%)
≥ USD 101,000	233 (15.43%)	151 (14.23%)	384 (14.94%)

* *p* ≤ 0.001 (Chi-square test), ^a^ Due to missing data, some categories do not add to 100% (*n* = 2571).

**Table 2:** Correlates of 30-day cigarette smoking, 30-day e-cigarette use, and cigarette and e-cigarette consumption rates (*n* = 2571).

Measures	30-Day E-Cigarette Use (Pop = Survey Respondents)	30-Day Cigarette Use (Pop = Survey Respondents)	E-Cigarette Consumption (Pop = E-Cigarette Users)	Cigarette Consumption (Pop = Cigarette Users)
	**OR (95% CI)**	**OR (95% CI)**	**IRR (95% CI)**	**IRR (95% CI)**
Pre/Post-lockdown order				
March (pre)	1	1	1	1
May (post)	**0.66 (0.55, 0.78)**	0.96 (0.75, 1.23)	1.03 (0.9, 1,13)	**1.13 (1.15, 1,23)**
Sex				
Male	1	1	1	1
Female	0.85 (0.71, 1.02)	**0.60 (0.47, 0.77)**	**0.84 (0.73, 0.97)**	1.06 (0.97, 1.14)
Age				
≥26 years old	1	1	1	1
18–25 years old	**1.75 (1.35, 2.27)**	0.97 (0.70, 1.34)	**0.78 (0.65, 0.94)**	1.03 (0.92, 1.15)
Race/ethnicity				
NH White	1	1	1	1
Hispanic/Latino	**0.29 (0.23, 0.37)**	**0.19 (0.14, 0.24)**	1.2 (0.99, 1.46)	1.06 (0.95, 1.19)
NH African-American/Black	**0.27 (0.19, 0.39)**	**0.44 (0.28, 0.68)**	1.26 (0.92, 1.73)	1.02 (0.88, 1.18)
NH Other	**0.43 (0.31, 0.61)**	**0.26 (0.17, 0.38)**	1.11 (0.85, 1.45)	0.96 (0.81, 1.13)
Lives with partner				
No	1	1	1	1
Yes	**1.83 (1.50, 2.24)**	**1.68 (1.29, 2.20)**	**1.22 (1.04, 1.42)**	**1.1 (1.01, 1.2)**
Education				
College degree or higher	1	1	1	1
Some college	1.11 (0.92, 1.36)	1.21 (0.91, 1.59)	**0.41 (0.34, 0.49)**	1 (0.89, 1.13)
High school/GED or less	**2.49 (1.86, 3.33)**	1.33 (0.92, 1.92)	**0.74 (0.64, 0.85)**	**0.91 (0.84, 1)**
Income				
≥USD 101,000	1	1	1	1
USD 76,000–USD 10,000	**0.69 (0.53, 0.91)**	**0.50 (0.31, 0.82)**	1.19 (0.98, 1.45)	**1.11 (0.99, 1.25)**
USD 51,000–USD 75,000	0.81 (0.62, 1.07)	**0.26 (0.16, 0.42)**	0.91 (0.76, 1.1)	**1.13 (1.01, 1.27)**
USD 0–USD 50,000	0.83 (0.62, 1.11)	**0.36 (0.22, 0.58)**	**0.7 (0.57, 0.87)**	**1.17 (1.03, 1.32)**
Constant	**1.54 (1.14, 2.07)**	**23.18 (13.75, 39.08)**	**125.63 (100.5, 157.05)**	**182.38 (159.84, 208.09)**

Note: Bolded items are significant at *p* ≤ 0.05. OR = odds ratio; IRR = incidence rate ratio; NH = Non-Hispanic; Pop = population.

## Data Availability

The data presented in this study are available on request to the corresponding author. The data are not publicly available since the dataset contains geographical information below the level of a US state.
